# Sudden intrauterine unexplained death: time to adopt uniform postmortem investigative guidelines?

**DOI:** 10.1186/s12884-019-2603-1

**Published:** 2019-12-30

**Authors:** Anna M. Lavezzi, Francesco Piscioli, Teresa Pusiol, Gianfranco Jorizzo, Stefano Ferrero

**Affiliations:** 1 “Lino Rossi” Research Center for the study and prevention of unexpected perinatal death and SIDS, Department of Biomedical, Surgical and Dental Sciences, University of Milan, Via della Commenda 19, 20122 Milan, Italy; 2 Institute of Pathology, Hospital of Rovereto (Trento), Rovereto, Italy; 3 Prenatal Medicine Service, Aulss 6 Euganean Territory, Padua, Italy; 4 Division of Pathology, Fondazione IRCCS Ca’ Granda, Ospedale Maggiore Policlinico, Milan, Italy

**Keywords:** Unexplained stillbirth, Fetal autopsy, Guidelines, Neuropathological protocol, Brainstem, Immunohistochemistry, Nicotine, Endocrine disrupting compounds (EDCs)

## Abstract

**Background:**

Worldwide approximately 2.6 million are stillborn, mostly occurring in developing countries. In the great part these deaths are inexplicable. The evenness and standardisation of the diagnostic criteria are prerequisites to understand their pathogenesis. The core goal of this article is to propose new evidence based investigative post-mortem guidelines that should be adopted in all the Institutions especially when a fetal death, after a routine autopsy procedure, is diagnosed as “unexplained”. The proposed protocol is mainly focused on the anatomopathological examination of the autonomic nervous system and in particular of the brainstem where the main centers that control vital functions are located.

**Methods:**

Updated investigative guidelines for the examination of unexplained stillbirths, prevalently focused on the histological examination of the brainstem, where the main centers that are involved in monitoring the vital functions are located, are here presented. A section of this protocol concerns the Immunohistochemical evaluation of specific functional markers such as the neuronal nuclear antigen, nicotinic acetylcholine receptors, serotonin, orexin, apoptosis and gliosis. The important role of risk factors, having regard in particular to maternal smoking and air pollution is also contemplated in these guidelines.

**Results:**

Specific morphological and/or functional alterations of vital brainstem structures have been found with high incidence in over 100 cases of unexplained fetal death sent to the “Lino Rossi Research Center” of the Milan University according to the Italian law. These alterations were rarely detected in a group of control cases.

**Conclusions:**

We hope this protocol can be adopted in all the Institutions notably for the examination of unexplained fetal deaths, in order to make uniform investigations. This will lead to identify a plausible explanation of the pathogenetic mechanism behind the unexplained fetal deaths and to design preventive strategies to decrease the incidence of these very distressing events for both parents and clinicians.

**Trial registration:**

not applicable for this study.

## Background

Fetal deaths, particularly at or near term, are the most common adverse pregnancy outcomes and the leading contributor to perinatal mortality. In 2015 there were 2.6 million stillbirths globally, with more than 7178 deaths a day. The majority of these deaths occurred in developing countries. The World Health Organization (WHO) describes a rate in sub-Saharan Africa approximately 10 times that of developed countries (29 vs. 3 per 1000 births). Other sources are providing similar epidemiology with a rate of about 5 per 1000 in developed countries [1–3].

Obviously, parents want to know why their baby died in the womb and the chance of recurrence in future pregnancies. Therefore, the detection of the pathogenetic mechanism leading to stillbirth is very important, not just to give an answer to parents, but also to introduce preventive measures in order to lower the perinatal mortality.

An important first step in case of stillbirth consists in performing an accurate autopsy, comprehensive of placenta and umbilical cord examination [4, 5]. A thorough obstetric and maternal history should also be taken, including exposures to risk factors.

In many cases a cause of death, attributable to fetal, maternal, or placental pathology, is clearly identified. However, after a careful investigation, one-half to two-thirds of stillbirths are still listed as death due to undeterminable reasons. From the examination of international literature, defects in the development of the autonomic nervous system (ANS) appear increasingly involved in the pathogenesis of sudden and unexplained intrauterine deaths. These deaths, called “unexplained stillbirths” [6, 7], should be considered as a syndrome and referred with the acronym “SIUDS”, i.e., “Sudden Intrauterine Unexplained Death Syndrome”, like to “SIDS” (Sudden Infant Death Syndrome) [8]. This suggested definition is based on the realization that several conditions, simultaneously occurring, may contribute to stillbirth and that unexplained stillbirths and SIDS share common brain abnormalities, associated with the same risk factors. So, first of all, to try to understand the pathogenesis of SIUDS, it is essential that the diagnostic criteria used by investigators are standardized and provide for the inclusion of the ANS examination. Above all, the deep anatomopathological study of the brainstem could highlight the presence of developmental alterations of specific nuclei that control the vital functions. This would allow to identify a common denominator in SIUDS cases, so providing a plausible explanation of the pathogenetic death mechanism. However, at this moment, the lack of uniform post-mortem protocols for evaluating stillbirths has hindered significant studies in this field.

The core goal of this article is to propose innovative evidence based investigative post-mortem guidelines that include, in particular, the in-depth examination of the autonomic nervous system (ANS) that has been developed by the “Lino Rossi- Research Center for the Study and Prevention of the Sudden Perinatal Death and SIDS” of the Milan University, in Italy, according to the directives of the Italian law 31/2006 “Regulations of diagnostic post mortem investigation in the victims of SIDS and unexpected fetal death” [9] . The neuropathological examination of nerve centers controlling vital functions, mostly sited in the brainstem, is a fundamental part of this protocol that, to the best of our knowledge, is the first drafted for this purpose. The role of exogenous risk factors in the genesis of neuronal damages is also considered.

## Methods

### A) Checklist of NEUROPATHOLOGICAL procedures for the examination of the brainstem in SIUDS

### Histopathological protocol

**Fig. 1:**
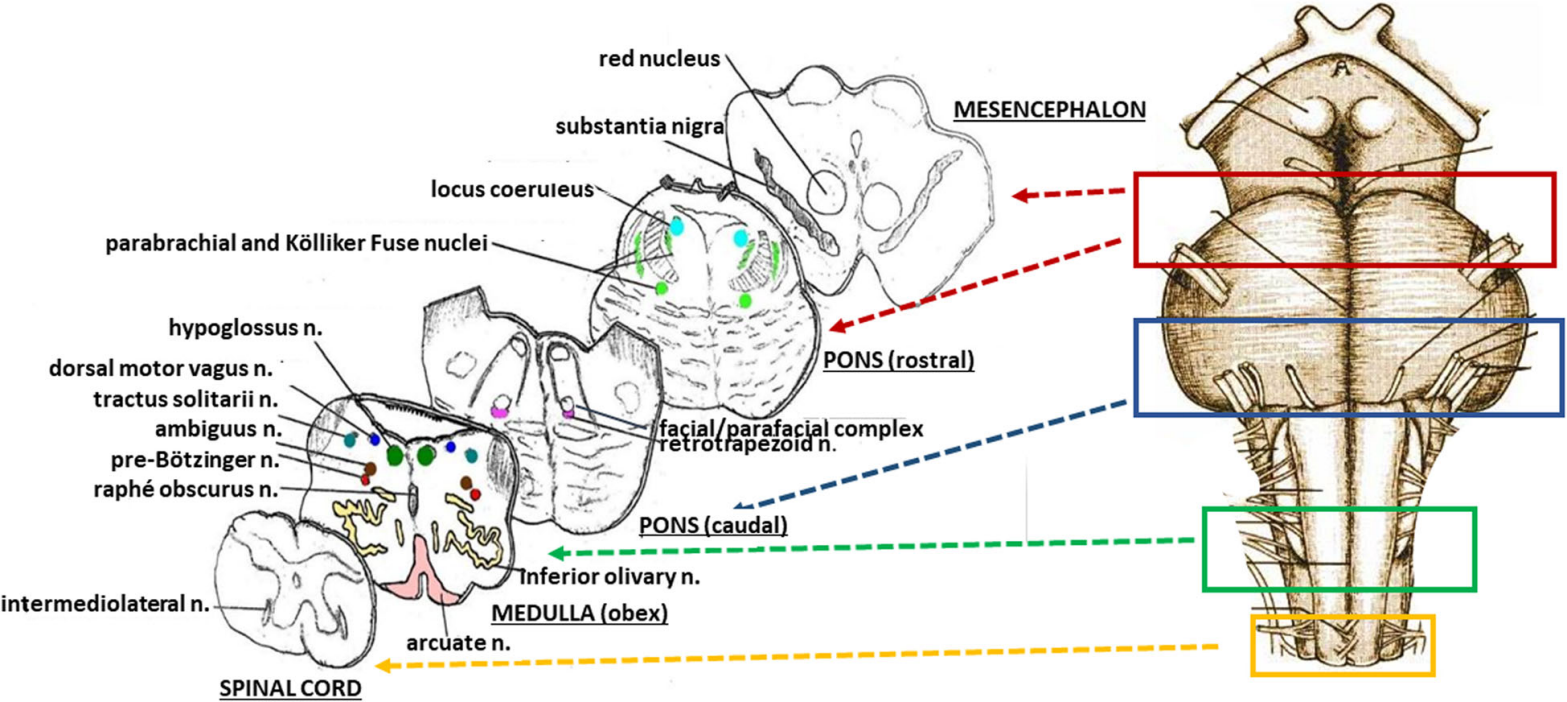
*At the right*, sampling of three specimens from the brainstem. The first specimen, ponto-mesencephalic, includes the upper third of the pons and the adjacent portion of mesencephalon. The second extends from the upper portion of the medulla oblongata to the adjacent caudal portion of the pons. The third specimen extends 2–3 mm above and below the obex. A fourth sample is taken from the rostral tract of spinal cord. *At the left*, the histological sections obtained from the specimens are represented, indicating the main nuclei and structures to be examined

A scheme of the methodology for the brainstem examination is represented in Fig. 1. At the right, the sampling of four specimens is shown. The first specimen includes the upper portion of the pons until the caudal mesencephalon. The second is focused essentially on the caudal pons. A third sample is taken from the medulla oblongata in correspondence of the obex. A fourth specimen extends from the caudal medulla to the rostral spinal cord.

### Technical details

**Fig. 2:**
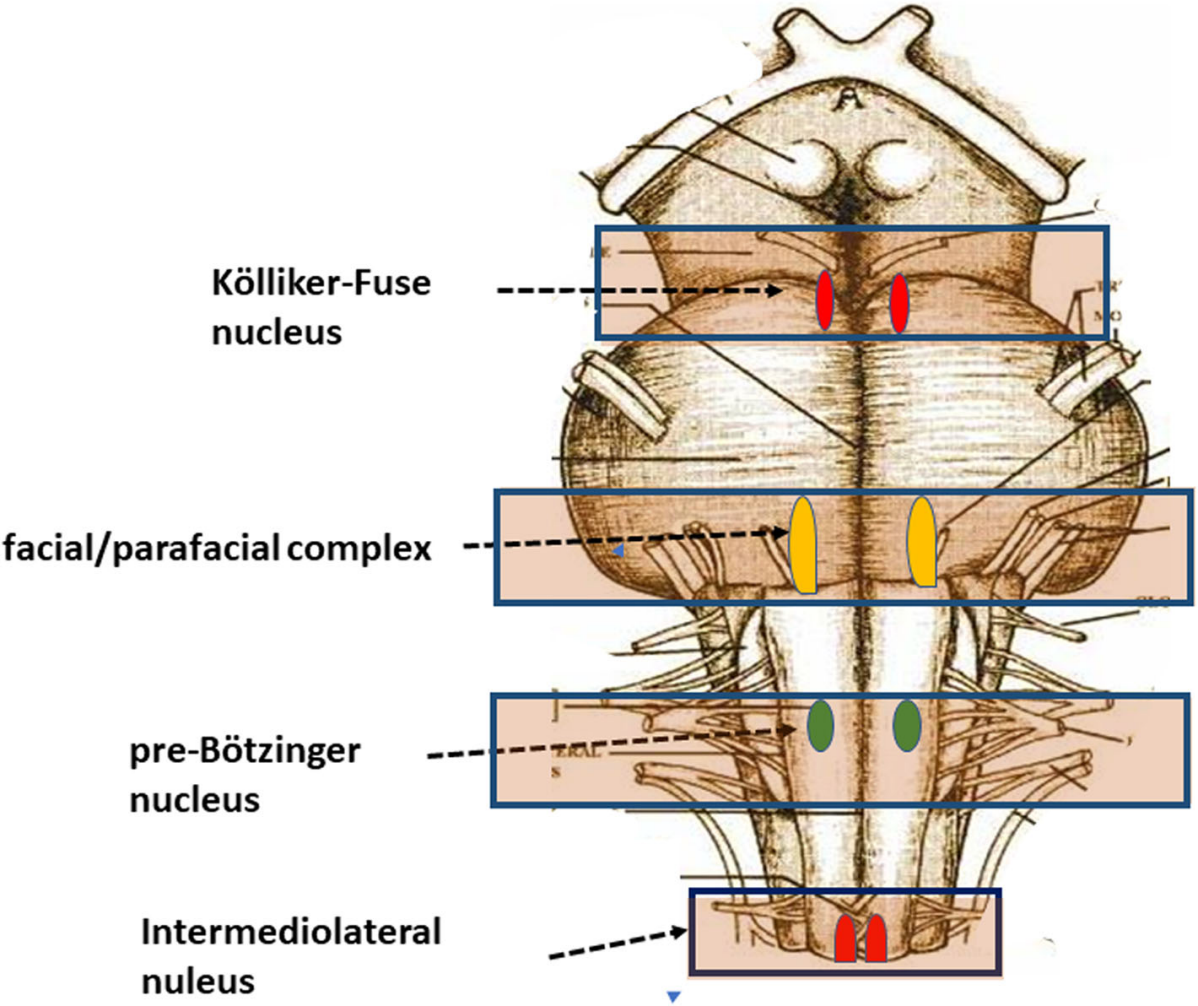
Localization and extension of the most important centers, components of the RN, in the brainstem samples

**Fig. 3:**
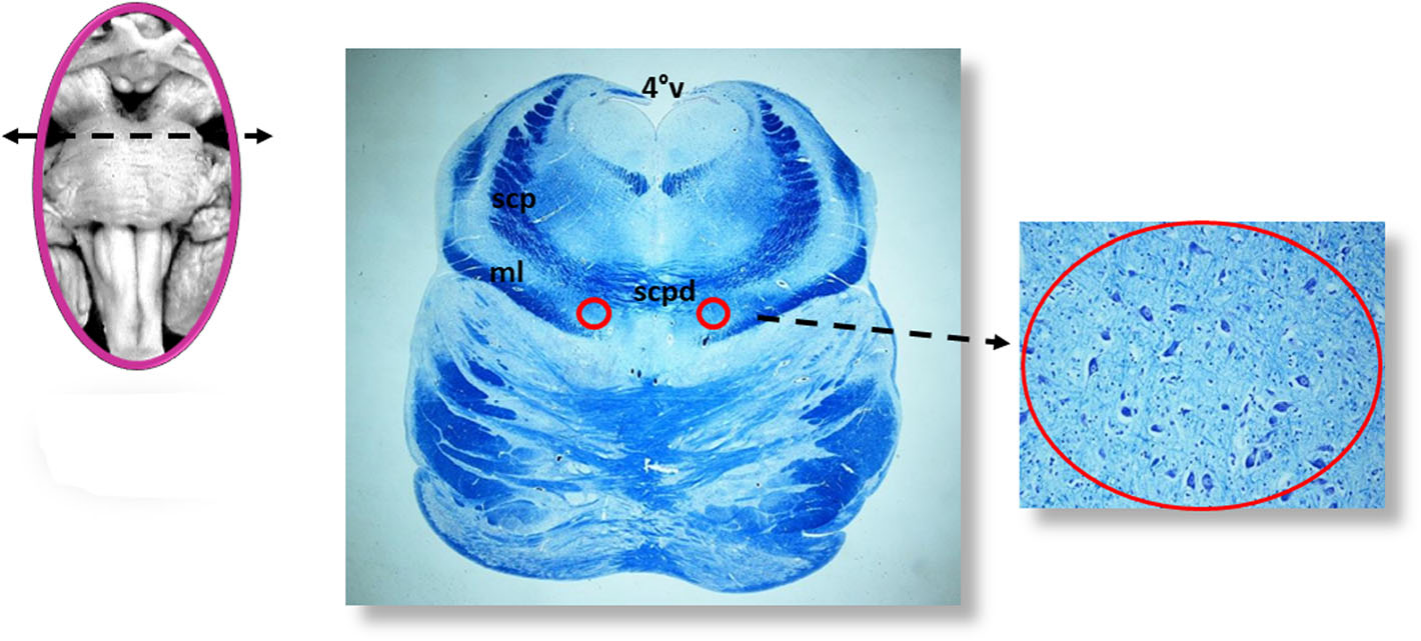
Kölliker-Fuse nucleus (red circle). scpd: decussation of upper cerebellar peduncles; scp: superior cerebellal peduncle; ml: medial lemniscus; 4°V: fourth ventricle. Staining of histological sections: Klüver-Barrera

**Fig. 4:**
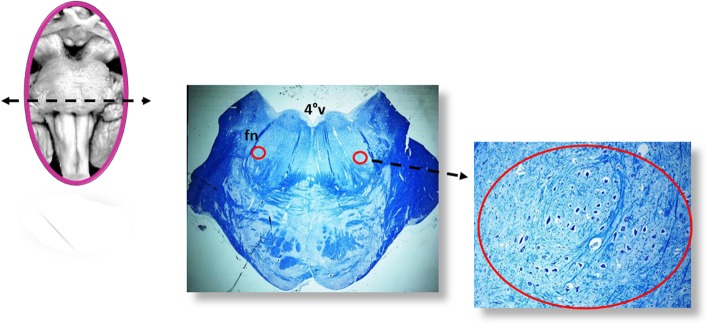
Facial/parafacial complex (red circle). fn: facial nerve; 4°V: fourth ventricle. Staining of histological sections: Klüver-Barrera

**Fig. 5:**
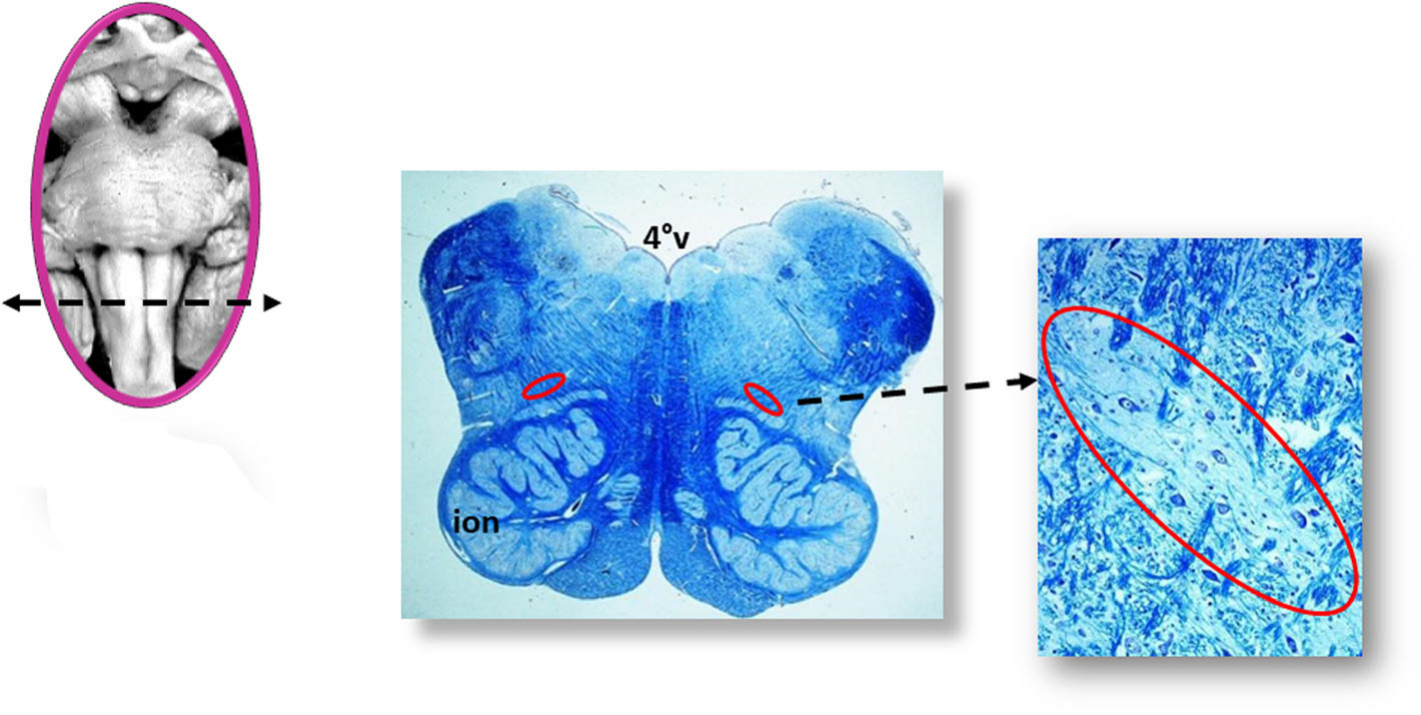
pre-Bötzinger nucleus (red circle). ion: inferior olivary nucleus; 4°V: fourth ventricle. Staining of histological sections: Klüver-Barrera

**Fig. 6:**
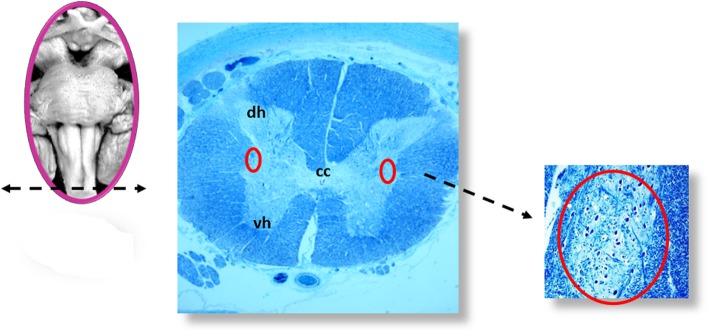
Intermediolateral nucleus (red circle). dh: dorsal horn; vh: ventral horn; cc: central canal; Staining of histological sections: Klüver-Barrera

All the samples are transversely cut every 60 μm. Serial histological sections, 5 μm in thickness, are carried out at each level. Two of these sections are at first stained by hematoxylin-eosin and Klüver-Barrera and examined by using a light microscope with a system of objective lenses to progressively magnify the images. Figure 1, at the left, shows the representative histological sections obtained from the above-described specimens, indicating the main nuclei and structures to be examined, given their frequent involvement in SIUDS in terms of delayed development (hypoplasia/agenesis). These nerve centers are more easily recognizable in histological sections stained with the Klüver-Barrera method. The Cresyl Violet technique can be used additionally to highlight the nuclear tigroid substance (Nissl bodies). The Gless-Marsland, consisting in a silver impregnation, can be applied to stain axons and dendrites. Essentially, the centers represented in Fig. 1, at the left, are: hypoglossus, dorsal motor vagus, tractus solitarii, ambiguus, inferior olivary, pre-Bötzinger, arcuate, obscurus raphé nucleus in the medulla oblongata; locus coeruleus, facial/parafacial complex, retrotrapezoid and Kölliker-Fuse nuclei in the pons; substantia nigra, and red nucleus in the mesencephalon. In the spinal cord the intermediolateral nucleus is of great interest. The histological examination must be focused especially on the Kölliker-Fuse nucleus, the facial/parafacial complex the pre-Bötzinger nucleus and the intermediolateral nucleus since these nerve structures are binded to each other through multiple synapses between their neurons in order to form a network which, through excitatory and/or inhibitory stimulations in relation to the need, is able to control breathing both before and after birth. This network is just called “respiratory network” (RN). Despite being so important, these four centers have a short extension and are completely included in the sampling carried out (Fig. 2). Figures 3, 4, 5 and 6 show the cytoarchitecture of these structures in histological sections and the level from which they were taken.

The RN must always be examined in fetuses, even if breathing is not a vital condition in utero. After all, one of its components, the intermediolateral nucleus, is recognized as the primary center used to mediate the occasional respiratory movements aimed to promote the fetal lung development. However, it is not easy to justify a fetal death associated to developmental alterations of the RN. Nevertheless, we can hypothesize that a verification of essential centers for extrauterine life occurs at the final phase of pregnancy. Whenever one of these centers, especially if involved in respiration control, is not well developed and therefore not fully functional, the fetus, without a seemingly understandable reason, eliminates itself in order to avoid, especially to parents, a much more tragic of a newborn death [10].

**Fig. 7:**
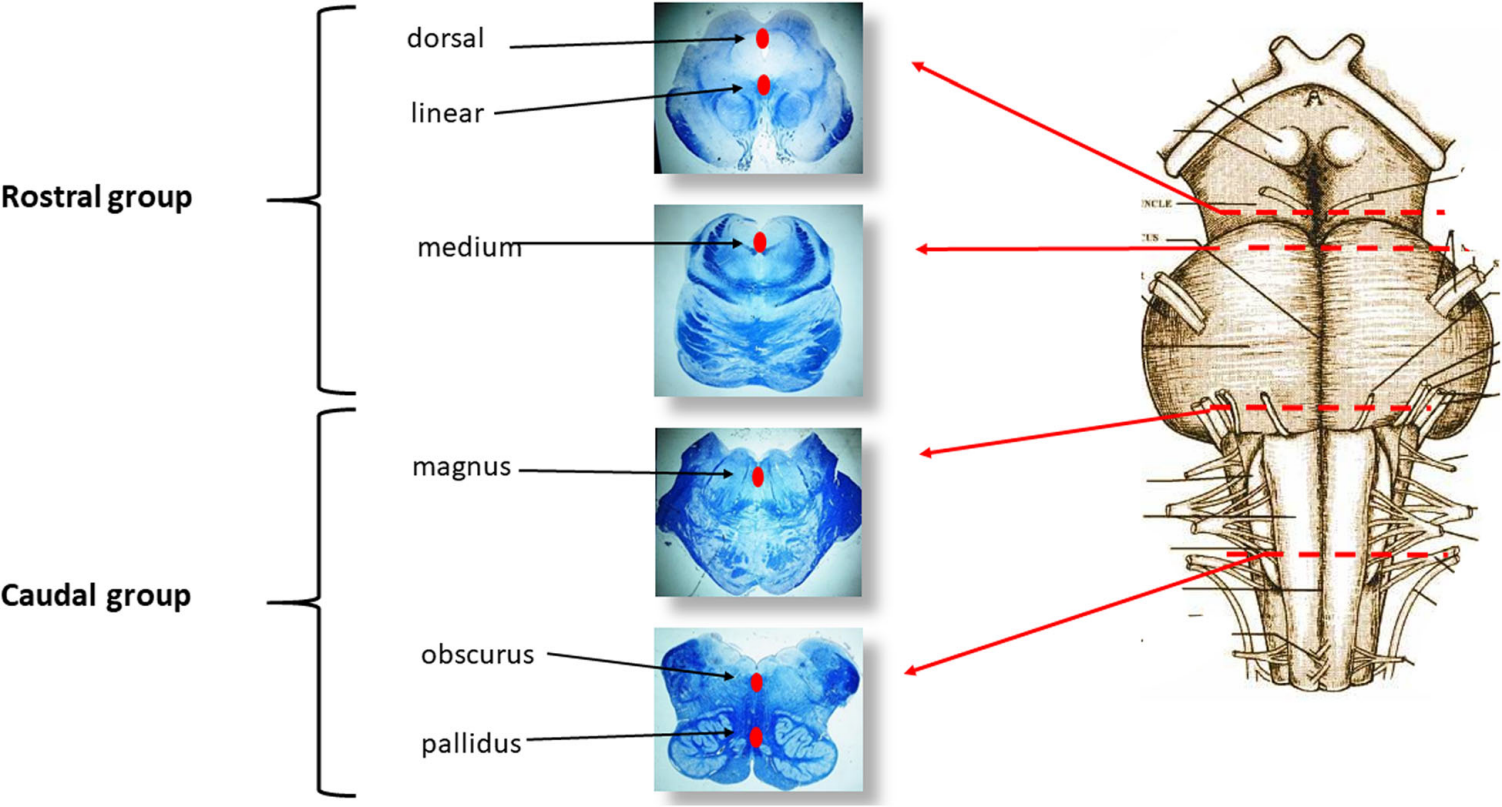
Raphé nuclei and their subdivision into two groups (rostral and caudal groups)

Further structures to be analyzed are the chemoreceptorial ones as they participate in the physiological control of respiration. The chemoreceptorial centers are able to detect the concentrations of gas and hydrogen in the interstitial fluid and to send information to the RN centers which consequently modulate their activity to maintain these parameters within normal values [11]. Among the numerous chemoreceptorial structures we point out the raphé system, a series of nuclei. located in the midline of the brainstem. They have been gathered in two groups: 1) the “rostral serotonergic raphé group”, confined to the mesencephalon and rostral pons (including the caudal linear raphé nucleus, the dorsal raphé nucleus, the median raphé nucleus) and 2) the “caudal serotonergic raphé group”, extending from the caudal pons to the caudal portion of the medulla oblongata (including the raphé magnus nucleus, the raphé obscurus nucleus and the raphé pallidus nucleus). Figure 7 shows the location of these nuclei in histological sections taken from the brainstem samples. The raphé system neurons produce serotonin, a neurotransmitter involved in numerous functions and especially in the control of respiration.

### Immunohistochemical protocol

Further sections obtained from the specimens are treated, according to the needs, with specific immunohistochemical techniques in order to evaluate functional markers whose expression is frequently altered in fetal deaths. Among these biological indicators are worthy of mention: the neuronal nuclear antigen, the nicotinic acetylcholine receptors, the serotonin, the orexin, the apoptosis and gliosis.

- **Neuronal nuclear antigen (NeuN)**

**Fig. 8:**
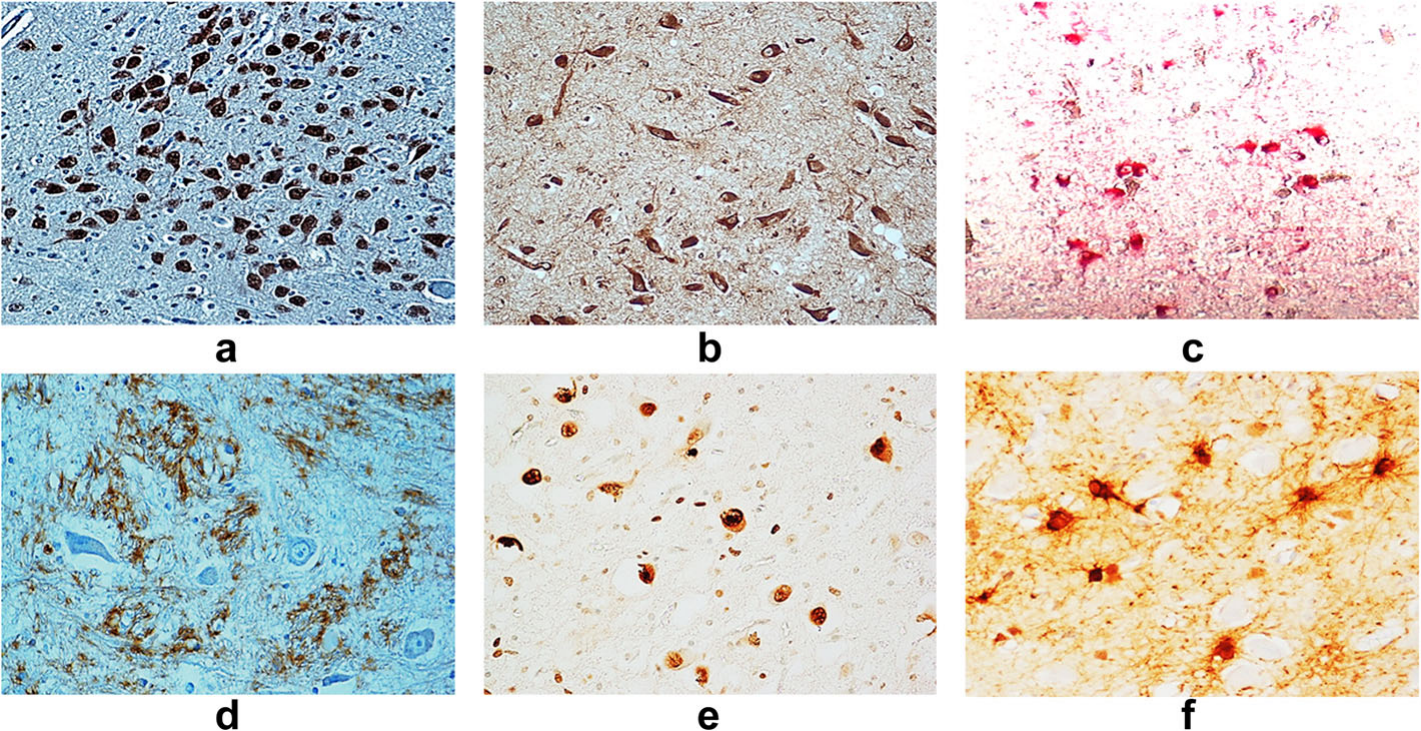
Immunohistochemical stains specific for different functional markers. Positive immunoexpression for: (**a**) NeuN; (**b**) nAChR; (**c**) 5-HT; (**d**) Ox; (**e**): apoptosi; (**f**) gliosi

This antigen shows normally a strong nuclear expression in post-mitotic healthy neurons even in prenatal life (Fig. 8A). A decreased immunopositivity of this antigen can be found in fetal brain as consequence of severe injuries, such as hypoxia, and can be indicative of neuronal degeneration in SIUDS [12].

- **Nicotinic acetylcholine receptors (nAChRs)**

The neuronal nicotinic acetylcholine receptors (nAChRs), a group of receptors which resulted from combination of different subunits, serve to mediate, through synaptic mechanisms, the transport of acetylcholine (ACh), the major cholinergic neurotransmitter which has a fundamental trophic role during brain development. These receptors show a cytoplasmic immunopositivity (Fig. 8B). The nAChRs can be activated not only by the ACh, but also by nicotine, (hence the name “nicotinic”). In case of a smoking mother in pregnancy, nicotine, once it has passed the blood-brain barrier of the fetal brain, can mimic the effect of Ach, since its active form is very close to Ach, and incorrectly promote the cholinergic activity of the nAChRs, so leading to neuronal damages [13–15].

- **Serotonin (5-HT)**

Serotonin (5-hydroxytryptamine) is a fundamental neurotransmitter mainly involved in the developmental process of neural vital circuits. It is synthesized, as previously reported, by the neurons of the raphé system (Fig. 8C) [16, 17].

- **Orexin**

Numerous studies to date have been focused on the important role of the orexin, a neuropeptide synthesized by neurons of the lateral hypothalamus, in the regulation of the sleep-wake state in infants and in its implications in SIDS pathogenesis, which, as well known, occurs in most cases at awakening from sleep [18]. Its immunopositivity is prevalently expressed in neuronal processes (Fig. 8D). Since the orexin system develops during the third trimester of pregnancy, with widespread connections from hypothalamic neurons to various neurotransmitter circuits, this neuropeptide could have additional important regulatory roles in the perinatal period. Then, it is advisable to perform also the immunohistochemical detection of orexin in intrauterine deaths.

- **Apoptosis and gliosis**

The application of immunohistochemical techniques for the study of apoptosis (TUNEL method) and glial fibrillary acid protein (GFAP) can be very useful to obtain information about the presence of brain cell death over the physiological levels, and of reactive gliosis, a process indicative of neuronal degeneration in SIUDS (Fig. 8E and F) [19, 20].

### B) Toxicological protocol

For understanding the SIUDS pathogenesis it is very important to collect information particularly related to risk factors such as maternal smoking, maternal alcohol and drug abuse and air pollution in the area where the mother lives. So, for each case of unexplained intrauterine death, all available information about pregnancy and fetal development, besides information related to the potential risk factors must be collected and categorised during post-mortem family interviews.

### -Main risk factors for unexplained fetal death

- **Nicotine**

Exposure to maternal tobacco smoke during pregnancy is associated with intrauterine growth retardation, abruptio placentae, low birth weight and a significantly higher risk of perinatal mortality [21, 22]. Mothers of stillbirths must be asked to report on their smoking habit before and during pregnancy. In addition, the removal of a lock of victims’ hair during the autopsy is needed to perform the toxicological search for the cotinine, the main metabolite of nicotine characterized by a long half-life. This test is aimed especially at verifying the negative assertions of the mothers. It is well known in fact that the retrospective assessment of maternal smoking, if performed after the fatal event, is sometimes unreliable because of feelings of guilt [23, 24]. In case of a smoker’s mother during pregnancy, nicotine and carbon monoxide (CO), its main combustion product, pass through the placenta into the fetal circulation where they can reach concentrations even 4 times higher than those present in maternal blood, due to the poor metabolic capacity of the fetal liver. The consequences can be multiple in the fetus. First of all, the carboxyhemoglobin, resulted by the binding of CO with hemoglobin, inhibits the release of oxygen into fetal tissues, so causing hypoxia especially in the most susceptible organs, including the brain. In addition, nicotine, being one of the few fat-soluble substances able to easily pass the blood-brain barrier by passive diffusion, giving its high affinity for nicotinic (acetylcholine) receptors as previously indicated, promptly binds to them, thus preventing the regular transmission of acetylcholine. It can also directly interfere with the expression of genes involved in the development of the nervous system, also inducing molecular alterations in DNA, RNA, and antigenic proteins of the neurons [25–27].

- **Pesticides**

The involvement of persistent pollutants such as pesticides and insecticides, a category of harmful agents belonging to the “endocrine disrupting compounds” (i.e. exogenous substances able to alter the functions of the endocrine system and, consequently, to affect the whole organism) has been highlighted in SIUDS [28–30]. Traces of highly toxic chemicals, as organochlorine and organophosphate pesticides (α and γ-chlordane, chlorfenvinfos, chlorpyrifos, p,p-DDT, p,p-DDE, endrin, α- and β-endosulfans) have been directly detected in brain samples of fetuses died in agricultural areas where they are used. These findings testify the possibility that such toxicants, like nicotine, easily pass the placental barrier into the fetus and then, through the blood brain barrier, into the fetal brain, so interfering with normal ANS development.

### C) Genetic analyses

Genetic investigations are an important component of fetal autopsies, not only in cases of congenital malformations, but also in case of unexplained intrauterine death. The use of the Polymerase Chain Reaction (PCR) is recommended in order to identify genes involved in neuronal dysgeneses. In particular, the serotonin transporter gene (*5-HTT*), the regulator of the synaptic serotonin concentration, the *PHOX2B* gene, a transcriptional factor involved in Congenital Central Hypoventilation Syndrome (CCHS) and the mitochondrial DNA (*mtDNA*), an important indicator of the cellular metabolisms, should be evaluated in SIUDS as they can offer important information on the pathogenetic mechanism of sudden death [17, 31].

## Results

The aforementioned guidelines have been already applied to a wide case series of SIUDS, sent to the “Lino Rossi Research Center” of the Milan University, according to the Italian law 31/2006 [9].

**Study population**- A cohort of 104 SIUDS (43 females and 61 males, 26–40 gestational weeks) and a group of 44 control cases (25 females and 19 males, 29–39 gestational weeks), consisting of intrauterine deaths due to a precise cause, specially collected and examined for comparison purposes, have been included in this study. Many of these cases have been the subject of our previous publications.

The findings, presented here, summarize all those obtained in numerous studies conducted over many years of research and which are the subject of 107 articles published in professional and peer-reviewed journals (19 of which are mentioned here in the References). The application of our neuropathological protocol allowed to identify in SIUDS high frequencies of specific developmental alterations prevalently of components of the RN. These defects were, on the contrary, rarely present in controls. Table 1 shows the incidence and the distribution of these alterations.

**Table 1:** Overall neuropathological brainstem findings in 104 SIUDS and 44 controls

Brainstem structure	Alterations		SIUDS n (%)	CONTROLS n (%)
	*classification*	*specific*		
Kölliker-Fuse nucleus	Morphological	hypoplasia, agenesis	61 (59%)	1 (2%)
	functional	<NeuN expression <Orexin expression	35 (34%) 31 (30%)	8 (18%) 2 (5%)
facial/ parafacial complex	morphological	hypoplasia, agenesis, neuronal immaturity	73 (70%)	3 (7%)
	functional	<NeuN expression < nAChRs	39 (37%) 41 (39%)	4 (9%) 0 (0%)
pre-Bötzinger nucleus	morphological	hypoplasia, agenesis, neuronal immaturity, <dendritic number	44 (42%)	0 (0%)
	functional	< NeuN expression < nAChRs	62 (60%) 44 (42%)	2 (5%) 0 (0%)
intermediolateral nucleus	morphological	hypoplasia, agenesis	57 (55%)	2 (5%)
	functional	reactive gliosis >apoptosis	45 (43%) 34 (33%)	0 (0%) 4 (9%)
raphé nuclei	morphological	hypoplasia, agenesis^a^	72 (69%)	5 (11%)
	functional	< serotonin expression^a^ < nAChRs reactive gliosis	55 (53%) 35 (34%) 64 (62%)	2 (5%) 0 (0%) 5 (11%)

^a^These alterations were significantly related to 5-HTT polymorphism (L/S and L/L genotypes). This association was detected overall in 45 SIUDS cases (43%)

Below, we indicate briefly the most frequent alterations we have highlighted in SIUDS.

### Neuropathologic findings in SIUDS

- **Morphological alterations**

We observed hypodevelopments (hypoplasia/agenesis/delayed neuronal maturation) of different nervous centers, mainly components of the RN, and precisely:

- hypoplasia, with a few immature neurons, or agenesis of the Kölliker-Fuse nucleus in the rostral pons [32, 33];
- hypoplasia of the facial/parafacial complex, with decreased neuronal density and cell body size in the caudal pons [34];
- hypoplasia of the pre-Bötzinger nucleus, with decreased cell and/or dendritic number in the medulla oblongata [35];
- various hypodevelopment degrees of the intermediolateral nucleus (neuronal immaturity in a normal structure/hypoplasia/agenesis) in the spinal cord [36].
- hypoplasia/agenesis of the raphe nuclei, especially of the raphe obscurus nucleus in the medulla oblongata.

Furthermore, hypodevelopment of other brainstem nuclei (hypoplasia of hypoglossal, dorsal vagal, tractus solitarii, inferior olivary nuclei) was occasionally observed.

- **Functional alterations**

Using specific immmunohistochemical methods we highlighted:

- total loss of immunoreactivity or decreased neuronal expression of NeuN antigen in the great part of the brainstem centers [37];
- decreased serotonin immunoexpression in the neuronal cell bodies and fibers of the raphé nuclei [17];
- altered expression of nAChRs in brainstem nuclei and/or neuronal complexes with both normal and delayed maturation [38];
- decreased presence of Ox- immunoreactive fibers especially around the Kölliker-Fuse neurons [39];
- unusual widespread apoptosis and high number of reactive astrocytes [40, 41].

Frequently, two or more morphological and/or functional alterations were simultaneously present in the same case.

Interesting is the close correlation that has been detected between hypoplasia of one or more nuclei of the raphé system, decreased serotonin expression and 5-HTT polymorphisms (L/L and/or L/S genotypes) [17].

The evaluation of the risk factors has highlighted a very significant correlation between neuropathological findings and maternal smoking in pregnancy [40, 42–44].

## Discussion

In case of intrauterine death, an accurate postmortem examination can reveal important information and findings that can highlight the possible causes of this inauspicious event. However, in the last decades, fetal autopsy rates have fallen to around 40–50%, despite the worldwide awareness of the need for adequate investigations above all in sudden unexplained fetal deaths. Furthermore, evidence supports that fetal autopsies must be performed by experienced perinatal pathologists, but the majority of hospitals do not have access to these specialists. In addition, the identification of specific causes of unexpected intrauterine deaths is hindered by the absence of a uniform investigative protocol.

In unexplained stillbirths, it is very important to perform a deep examination of the ANS as it can highlight subtle developmental alterations able to provide a plausible explanation for the death.

In 2011, the Stillbirth Collaborative Research Network in the United States has drawn up a detailed neuropathologic examination protocol specifically dedicated to determine lesions of the ANS and with the aim to highlight the cause of unexplained stillbirths [45]. This protocol however analyzes general parameters, such as brain weight related to gestational age, gyri and sulci structure in cerebral cortex, dendritic and axonal growth and onset and timing of myelination. No reference is made to developmental alterations of brain centers that coordinate the vital activities.

Here we propose a guideline model that is suitable for universal adoption. This is mainly designed to in-depth analyze the nerve centers located in the brainstem that are involved in monitoring essential functions and whose frequent alterations are very important in understanding the pathogenesis of intrauterine deaths. Our protocol is the result of many years of study performed at the “Lino Rossi” Research Center of the Milan University, which is the referral Center for the application of the Italian Law 31/2006 [9]. This law imposes in particular that all fetuses that die after 25th week of gestation without any apparent cause must be rapidly submitted to in-depth diagnostic post mortem investigation, following a tested diagnostic methodology.

The importance and value of these guidelines has been validated by many studies carried out by our Unit on over hundred cases of SIUDS, especially occurred in the last weeks of gestation, allowing to identify developmental morphological and/or functional alterations of vital nervous centers, mostly components of the RN. Impairment of respiratory centers have been frequently demonstrated in SIDS, often related to maternal cigarette smoking absorption during pregnancy, thus demonstrating a particular sensitivity of these structures to risk factors. While it is known that defective breathing control is the primary cause of sudden infant death, the question arises whether developmental alterations of respiratory centers can cause death during intrauterine life, when breathing is not yet a vital condition. We are unable to provide an answer, nevertheless we have formulated a hypothesis based on the observation that these alterations are mainly detected in fetuses that died at the end of pregnancy. Precisely, we suppose that approaching the birth, nature provides to check all the nervous structures that are essential for extrauterine life. When these are not fully developed and cannot therefore guarantee survival after birth, nature itself, in order to prevent such a nefarious event as the death of a newborn baby, decrees the fetal death.

Very interesting was the presence observed in almost half of SIUDS cases of polymorphisms of the 5-HTT gene, a gene normally involved in the synthesis, storage, membrane uptake, and metabolism of serotonin, associated with both morphological and functional defects of the raphé nuclei. The presence of a Long (L) allele (L/L and/or L/S genotypes) results in serotonin network dysfunction, and consequently in a failure of autonomic and respiratory responses. The association of 5-HTT polymorphism with SIDS has been widely documented in literature [46, 47]. The observation of 5-HTT polymorphisms in SIUDS could be relevant to propose targeted genetic tests to parents to alert them to a possible recurrence of a fetal death, where appropriate.

We believe that the examination of the central nervous system following our protocol, that includes histological, immunohistochemical, genetic investigations and, in addition, the evaluation of specific risk factors and their correlation with the neuropathological findings, should become a specialized component of the fetal autopsy above all when a clear cause of death is not found at routine examination.

We have also provided a data bank for collecting and storing all the information obtained from the application of our protocol, according to the highest standards of safety policies. The collected data will become available to all people interested in conducting studies and statistical researches that could provide the basis for appropriate prevention rules to reduce the SIUDS incidence.

Our guidelines will be presented at seminars and scientific conferences hoping that they can be implemented and disseminated.

## Conclusions

In case of unexplained fetal death, only the analysis of the multiple parameters and above all the application of the neuropathological protocol here presented, can allow to explain the possible pathogenetic mechanisms leading to death and consequently to plan effective prevention strategies. We propose to name our guidelines as “The Lino Rossi Protocol for investigating Causes of SIUDS”, with the hope that it can be accepted and applied worldwide.

## Abbreviations

5HT: 5-hydroxytryptamine (serotonin).
ANS: autonomic nervous system
mtDNA: mitochondrial DNA
NeuN: neuronal nuclear antigen
Ox: orexin
PCR: polymerase chain reaction
RN: respiratory network
SIDS: sudden infant death syndrome
SIUDS: sudden intrauterine unexplained death syndrome

## References

[1] Reinebrant HE, Leisher SH, Coory M (2018). Making stillbirths visible: a systematic review of globally reported causes of stillbirth. BJOG.

[2] World Health Organization. Maternal, newborn, child and adolescent health. Stillbirths. Geneva, Switzerland: WHO; 2016 (available at: www.who.int/maternal_child_adolescent/epidemiology/stillbirth/en/) .

[3] Wang H, Bhutta ZA, Coates MM, Coggeshall M, Dandona L (2016). Global, regional, national, and selected subnational levels of stillbirths, neonatal, infant, and under-5 mortality, 1980–2015: a systematic analysis for the global burden of disease study 2015. Lancet.

[4] Gordijn SJ, Erwich J, Khong TY (2002). Value of perinatal autopsy: critique. Pediatr Dev Pathol.

[5] Stock SJ, Goldsmith L, Evans MJ, Laing IA (2010). Interventions to improve rates of post-mortem examination after stillbirth. Eur J Obstet Gynecol Reprod Biol.

[6] Gardosi J, Kady SM, McGeown P, Francis A, Tonks A (2005). Classification of stillbirth by relevant condition at death (ReCoDe): population based cohort study. BMJ..

[7] Warland J, Mitchell EA (2014). A triple risk model for unexplained late stillbirth. BMC Pregnancy Childbirth.

[8] Matturri L, Pusiol T, Lavezzi AM (2014). Proposal of the acronym “SIUDS” for unexplained stillbirths, like “SIDS”. J Neonatal Biol.

[9] Constitution of the Italian Republic Law n° 31. Regulations for diagnostic post-mortem investigation in victims of sudden infant death syndrome (SIDS) and unexpected fetal death. Official Gazette of the Italian Republic, General Series 2006; 34: 4. Available at: http://users.unimi.it/centrolinorossi/files/gazz_ufficiale.pdf.

[10] Lavezzi AM, Ferrero S, Matturri L, Roncati L, Pusiol T (2016). Developmental neuropathology of brainstem respiratory centers in unexplained stillbirth: What's the meaning?. Int J Dev Neurosci.

[11] Nattie EE, Kaila K, Ransom BR (1998). Central chemoreceptors, pH and respiratory control. pH and brain function.

[12] Unal-Cevik I, Kilinç M, Gürsoy-Ozdemir Y, Gurer G, Dalkara T (2004). Loss of NeuN immunoreactivity after cerebral ischemia does not indicate neuronal cell loss: a cautionary note. Brain Res.

[13] Govind AP, Vezina P, Green WN (2009). Nicotine-induced Upregulation of nicotinic receptors: underlying mechanisms and relevance to nicotine addiction. Biochem Pharmacol.

[14] Lindstrom J, Anand R, Gerzanich V, Peng X, Wang F, Wells G (1996). Structure and function of neuronal nicotinic acetylcholine receptors. Prog Brain Res.

[15] Boyd RT (1997). The molecular biology of neuronal nicotinic acetylcholine receptors. Crit Rev Toxicol.

[16] Kinney HC, Belliveau RA, Trachtenberg FL, Rava LA, Paterson DS (2007). The development of the medullary serotonergic system in early human life. Auton Neurosci.

[17] Lavezzi AM, Casale V, Oneda R, Weese-Mayer DE, Matturri L (2009). Sudden infant death syndrome and sudden intrauterine unexplained death: correlation between hypoplasia of raphé nuclei and serotonin transporter gene promoter polymorphism. Pediatr Res.

[18] Sakurai T, Mieda M (2010). Tsujino. The orexin system: roles in sleep/wake regulation. Ann N Y Acad Sci.

[19] Martin LJ (2001). Neuronal cell death in nervous system development, disease and injury. Rev Int J Mol Med.

[20] Norenberg MD (1994). Astrocytes responses to CNS injury. J Neuropathol Exp Neurol.

[21] Reeves S, Bernstein I (2008). Effects of maternal tobacco-smoke exposure on fetal growth and neonatal size. Expert Rev Obstet Gynecol.

[22] Pineles BL, Hsu S, Park E, Samet JM (2016). Systematic Review and Meta-Analyses of Perinatal Death and Maternal Exposure to Tobacco Smoke During Pregnancy. Am J Epidemiol.

[23] Tzatzarakis MN, Vardavas CI, Terzi I, Kavalakis M, Kokkinakis M, Liesivuori J, Tsatsakis AM (2012). Hair nicotine/cotinine concentrations as a method of monitoring exposure to tobacco smoke among infants and adults. Hum Exp Toxicol.

[24] Jacqz-Aigrain E, Zhang D, Maillard G, Luton D, André J, Oury JF (2002). Maternal smoking during pregnancy and nicotine and cotinine concentrations in maternal and neonatal hair. BJOG.

[25] Chatterton Z, Hartley BJ, Seok MH, Mendelev N, Chen S, Milekic M (2017). In utero exposure to maternal smoking is associated with DNA methylation alterations and reduced neuronal content in the developing fetal brain. Epigenetics Chromatin.

[26] Lavezzi AM, Ottaviani G, Matturri L (2005). Adverse effects of prenatal tobacco smoke exposure on biological parameters of the developing brainstem. Neurobiol Dis.

[27] Lichtensteiger W, Ribary U, Schlumpf M, Odermatt B, Widmer HR (1988). Prenatal adverse effects of nicotine on the developing brain. Prog Brain Res.

[28] Roncati L, Termopoli V, Pusiol T (2016). Negative role of the environmental endocrine disruptors in the human neurodevelopment. Front Neurol.

[29] Roncati L, Pusiol T, Piscioli F, Lavezzi AM (2017). Neurodevelopmental disorders and pesticide exposure: the northeastern Italian experience. Arch Toxicol.

[30] Lavezzi AM, Cappiello A, Termopoli V, Bonoldi E, Matturri L (2015). sudden infant death with area postrema lesion likely due to wrong use of insecticide. Pediatrics..

[31] Colleoni F, Lattuada D, Garretto A, Massari M, Mandò C, Somigliana E, Cetin I (2010). Maternal blood mitochondrial DNA content during normal and intrauterine growth restricted (IUGR) pregnancy. Am J Obst Gynecol.

[32] Lavezzi AM, Ottaviani G, Ballabio G, Rossi L, Matturri L (2004). Preliminary study on the cytoarchitecture of the human parabrachial/Kölliker-fuse complex, with reference to sudden infant death syndrome and sudden intrauterine unexplained death. Pediatr Dev Pathol.

[33] Lavezzi AM, Ferrero S, Paradiso B, Chamitava L, Piscioli F, Pusiol T (2019). Neuropathology of early sudden infant death syndrome—hypoplasia of the Pontine Kolliker-fuse nucleus: a possible marker of unexpected collapse during skin-to-skin care. Am J Perinatol.

[34] Lavezzi AM, Matturri L (2008). Hypoplasia of the parafacial/facial complex: a very frequent finding in sudden unexplained fetal death. Open Neurosci J.

[35] Lavezzi AM, Matturri L (2008). Functional neuroanatomy of the human pre-Bötzinger complex with particular reference to sudden unexplained perinatal and infant death. Neuropathol..

[36] Lavezzi AM, Corna MF, Mehboob R, Matturri L (2008). Neuropathology of the intermediolateral nucleus of the spinal cord in sudden unexplained perinatal and infant death. Int J Dev Neurosci.

[37] Lavezzi Anna M., Corna Melissa F., Matturri Luigi (2013). Neuronal nuclear antigen (NeuN): A useful marker of neuronal immaturity in sudden unexplained perinatal death. Journal of the Neurological Sciences.

[38] Lavezzi AM, Cappiello A, Pusiol T, Corna MF, Termopoli V, Matturri L (2015). Pesticide exposure during pregnancy, like nicotine, affects the brainstem α7 nicotinic acetylcholine receptor expression, increasing the risk of sudden unexplained perinatal death. J Neurol Sci.

[39] Lavezzi AM, Ferrero S, Roncati L, Matturri L, Pusiol T (2016). Impaired orexin receptor expression in the Kölliker-fuse nucleus in sudden infant death syndrome: possible involvement of this nucleus in arousal pathophysiology. Neurol Res.

[40] Lavezzi AM, Ottaviani G, Mingrone R, Matturri L (2005). Analysis of the human locus coeruleus in perinatal and infant sudden unexplained deaths. Possible role of the cigarette smoking in the development of this nucleus. Brain Res Dev Brain Res.

[41] Matturri L, Lavezzi AM. Unexplained stillbirth versus SIDS: common congenital diseases of the autonomic nervous system. Pathology and nosology. Early Hum Dev. 2011;87:209–215. doi: 10.1016/j. 10.1016/j.earlhumdev.2010.12.009 21262556

[42] Lavezzi Anna M, Corna Melissa F, Matturri Luigi (2010). Ependymal alterations in sudden intrauterine unexplained death and sudden infant death syndrome: possible primary consequence of prenatal exposure to cigarette smoking. Neural Development.

[43] Lavezzi AM, Ottaviani G, Mauri M, Matturri L (2007). Biopathology of the dentate-olivary complex in sudden unexplained perinatal death and sudden infant death syndrome related to maternal cigarette smoking. Neurol Res.

[44] Lavezzi AM, Matturri L, Del Corno G, Johanson CE (2013). Vulnerability of fourth ventricle choroid plexus in sudden unexplained fetal and infant death syndromes related to smoking mothers. Int J Dev Neurosci.

[45] Pinar H, Koch MA, Hawkins H, Heim-Hall J, Shehata B, Thorsten VR, Chin S, Willinger M, dela Monte S (2011). the stillbirth collaborative research network neuropathologic examination protocol. Am J Perinatol.

[46] Weese-Mayer DE, Berry-Kravis EM, Maher BS, Silvestri JM, Curran ME, Marazita ML (2003). Sudden infant death syndrome: association with a promoter polymorphism of the serotonin transporter gene. Am J Med Genet A.

[47] Qin H, Xu G, Pan X, Mo Y (2016). Meta-analysis of the association between serotonin transporter polymorphisms and sudden infant death syndrome. J Forensic Sci Med.

